# C1 inhibitor deficient hereditary angioedema is related to endothelial dysfunction in young adult and middle‐aged patients

**DOI:** 10.1002/clt2.70076

**Published:** 2025-06-23

**Authors:** Gokce Gul Atay Sensoy, Derya Baykız, İlkim Deniz Toprak, Deniz Eyice Karabacak, Erdem Bektas, Derya Unal, Aslı Gelincik, Semra Demir

**Affiliations:** ^1^ Department of Internal Medicine Istanbul Faculty of Medicine Istanbul University Istanbul Turkey; ^2^ Department of Cardiology Istanbul Faculty of Medicine Istanbul University Istanbul Turkey; ^3^ Division of Immunology and Allergic Diseases Department of Internal Medicine Istanbul Faculty of Medicine Istanbul University Istanbul Turkey

**Keywords:** carotid intima‐media thickness, endothelial dysfunction, flow‐mediated dilation, hereditary angioedema

## Abstract

**Background:**

Hereditary angioedema (HAE) is a rare, autosomal dominantly inherited disease characterised by mucocutaneous oedema attacks. Vasoactive mediators and the endothelium play an important role in the pathogenesis of HAE. We aimed to evaluate the endothelial dysfunction in HAE.

**Methods:**

The study included 35 C1 inhibitor deficient (C1‐INH) HAE patients aged 18–50 years old without any risk factor that could cause endothelial dysfunction, and 25 sex‐ and age‐matched healthy controls (HCs). Bilateral carotid intima‐media thickness (CIMT), flow‐mediated dilation (FMD) measurements, and transthoracic echocardiography (ECHO) imaging were performed. Demographic and clinical features of the patients were evaluated.

**Results:**

The percentage of FMD (FMD [%]) in C1‐INH HAE patients was significantly lower than in the HCs (*p* < 0.001). There was no significant difference in the CIMT between C1‐INH HAE patients and HCs. In addition, the findings of the ECHO were similar between the groups. C4 and C1 INH levels at diagnosis, gender, age, disease severity, presence of long‐term prophylaxis treatment and attack frequency were not associated with FMD (%), whereas disease duration was correlated with lower FMD (%) (*r* = −0.480, *p* = 0.003).

**Conclusion:**

The present study indicated the presence of structural endothelial dysfunction in C1‐INH HAE in the absence of atherosclerosis. Moreover, the study revealed that endothelial dysfunction was associated with disease duration, irrespective of disease severity. Further studies are required in order to assess mortality and morbidity due to endothelial dysfunction in C1‐INH HAE and to determine the molecular mechanisms underlying endothelial dysfunction in C1‐INH HAE.

## 1 INTRODUCTION

Hereditary angioedema (HAE) is a rare autosomal dominantly inherited genetic disorder characterised by subcutaneous, submucosal, and mucosal tissue oedema attacks, in the absence of urticaria and/or pruritus.^1^ The oedematous attacks primarily affect the skin, abdomen, genitalia, and upper respiratory tract, with mortality potential due to asphyxia.^2^, ^3^ C1 inhibitor deficiency (C1‐INH) resulting from mutations in the SERPING1 gene is determined by low serum C1 INH level and function in type I C1‐INH HAE, whereas normal or high serum C1 INH concentration and low its function in type II C1‐INH HAE. Furthermore, HAE with normal C1 inhibitor levels (HAE‐nC1‐INH) resulting from various mutations, including kininogen‐1 (HAE‐KNG1), plasminogen gene (PLG‐HAE), myoferlin gene mutation (MYOF‐HAE), heparan sulphate‐glucosamine 3‐sulfotransferase 6 (HS3ST6), mutation in Hageman factor (factor XII), and in angiopoietin‐1 (HAE‐ANGPT‐1) has also been described.^4^, ^5^

The development of symptoms and signs in HAE is attributed to increased vascular permeability. Bradykinin, a member of the kallikrein–kinin system, a short‐lived and potent vasoactive peptide activated by the plasma contact system in C1‐INH deficiency or dysfunction, is the primary mediator responsible for increased vascular permeability and thus oedema in HAE. Bradykinin increases the vascular permeability by binding to B2 receptors, which are mainly expressed on the surface of endothelial cells. Following binding to its receptor, phosphorylation of transmembrane vascular endothelial cadherin molecules occurs. This subsequently increases the pore sizes in endothelial cells, which is a consequence of the actin cytoskeleton contraction. The result of this endothelial activation is the development of vascular leakage.^6^

Endothelial dysfunction is a predisposition of the endothelium to impaired vasodilatation capacity, a pro‐inflammatory status, and pro‐thrombotic tendencies.^7^ It has been linked to a variety of cardiovascular diseases, including hypertension, chronic heart failure, coronary artery disease, peripheral arterial disease, chronic renal failure, and diabetes.^8^ A reduction in nitric oxide synthesis, the presence of excessive oxidative products, and a reduction in the production of hyperpolarizing factor collectively contribute to the impairment of the vasodilator response.^9^ Endothelial dysfunction is a pathological process that develops prior to the onset of overt morphological changes and clinical symptoms. Therefore, its early detection seems essential for the prevention of future atherosclerosis related mortalities and morbidities. Flow mediated dilatation (FMD) assessment was recognised as a non‐invasive method to evaluate in vivo vasodilator function, which is an indicator of endothelial integrity. It is considered to reflect endothelium‐dependent and nitric oxide‐mediated arterial function.^10^

Endothelial dysfunction in HAE in which vasoactive mediators acting on the endothelium are involved in the pathogenesis has been evaluated in various studies.^11^, ^12^, ^13^ Herein, we aimed to investigate endothelial dysfunction in C1‐INH HAE in a population with a low risk of cardiovascular disease and no atherosclerosis.

## 2 METHODS

### 2.1 Patients and study design

This study enroled 35 patients between the ages of 18 and 50 years with a diagnosis of C1‐INH HAE who were being followed up at the Division of Immunology and Allergic Diseases in Istanbul University, Istanbul Faculty of Medicine. Patients had confirmed diagnosis of C1‐INH HAE according to current guideline.^3^ Patients who had conditions that may cause endothelial dysfunction such as diabetes mellitus (i.e., HbA1c ≥ 6.5%, fasting glucose level ≥126 mg/dL, and treatment on oral anti‐diabetic drugs or insulin), overt cardiovascular disease (i.e., coronary artery disease, cerebrovascular disease, and peripheral artery disease etc.), hypertension (i.e., treatment on anti‐hypertensive drugs, and systolic blood pressure >140 mmHg or diastolic blood pressure >90 mmHg), hyperlipidaemia (i.e., treatment on lipid lowering drugs, and dyslipidaemia), patients with abnormal findings in transthoracic echocardiography (ECHO) and patients who are active and/or passive smokers or obese were excluded from the study. Twenty‐five age‐ and sex‐matched individuals with a body mass index (BMI) of 20–25 kg/m^2^, and without conditions that could cause endothelial dysfunction were included as healthy controls (HCs).

Patients' demographics, clinical data, and laboratory findings were obtained from their medical records, and noted on standard forms. The number of HAE attacks in the last year was obtained from the patients' angioedema diary and emergency records. The HAE clinical severity score was determined according to the previous published data (Table S1).^14^

FMD assay was performed to assess endothelial dysfunction. Transthoracic ECHO was performed to evaluate the effect of possible valvular pathology and/or increased myocardial wall thickness on endothelial function. In addition, bilateral measurements of the carotid intima‐media thickness (CIMT) were performed in order to distinguish the atherosclerotic vascular disease from structural endothelial dysfunction. All measurements were conducted by a single experienced cardiologist (D.B.) who was blinded to previously obtained data in order to minimise the potential for inter‐ and intra‐observer variability.

This study protocol was approved by Istanbul University, Istanbul Faculty of Medicine, Clinical Research Ethics Committee (Date‐Number: 2020‐25469) and was in accordance with the standards set out in the Declaration of Helsinki. All subjects provided written informed consent.

### 2.2 Flow‐mediated vasodilation assay

Brachial artery FMD was evaluated non‐invasively according to the previously described procedure.^10^ Briefly, Brachial artery FMD measurements were performed using a Vivid 7 echocardiography system (General Electrics, Milwaukee, WI) using a 10 mm linear probe. The arm was maintained in a fixed position throughout the procedure, the measurement site was clearly marked, and the images were captured from the same location. The linear‐array transducer was placed 3–7 cm proximal of the antecubital fossa, and the baseline brachial artery diameter (BAD) was measured using longitudinal approach. Simultaneous electrocardiography was recorded, and the end‐diastolic BAD was measured with synchronised to the peak of the R wave. Subsequently, the aneroid sphygmomanometer cuff was wrapped around the forearm, and inflated at a pressure of 50 mmHg above the systolic blood pressure for a period of 5 min in order to induce ischaemia. Following the deflation of the cuff, and a 60‐s waiting period, the diameter of the brachial artery was measured, and the FMD response to reactive hyperaemia was evaluated. The BAD was measured three times both at baseline and after ischaemia, and the mean of the measurements was recorded. FMD (%) was calculated using the following formula.

$$\text{FMD}\;\left(\%\right)=100\times \left[\left({\text{BAD}}_{\text{hyperaemic}}-{\text{BAD}}_{\text{baseline}}\right)∕{\text{BAD}}_{\text{baseline}}\right]$$

### 2.3 Transthoracic echocardiography

All subjects were imaged in the left lateral decubitus position with a Vivid 7 ECHO system (General Electrics, Milwaukee, WI). Parasternal and apical images were obtained using sound waves in the mid‐level frequency range (3–8 MHz) (2D, M‐mode, Doppler Echocardiography). Transthoracic ECHO was performed as per the guidelines of the American Society of Echocardiography.^15^

### 2.4 Measurement of the carotid intima‐media thickness

CIMT was also evaluated non‐invasively accordance with a previously described procedure.^16^ Briefly, the patients were positioned in the supine position with their heads in extension for the measurement of CIMT. The right and left carotid arteries were visualised using the 10 MHz linear transducer of the Vivid 7 ECHO system (General Electrics, Milwaukee, WI). One centimetre segment was identified within the first 2 cm distal region from the common carotid artery bulb, and the thickness of the intima media was quantified by measuring the distance between the lumen of the vessel and the echogenic line formed by the media layer.

### 2.5 Statistical analysis

The quantitative variables were described as the mean with standard deviation or median with interquartile range, while the categorical variables were presented as a number and percentage. The Kolmogorov‐Smirnov test was performed to determine the normality of the data. Mann‐Whitney *U* test was used to compare quantitative variables according to the distribution of the data. Correlation analyses were conducted using Spearman's rho, given that the relevant variables were not normally distributed in at least one group. All analyses were performed using SPSS version 26 (IBM Corp), and a two‐tailed *p*‐value <0.05 was considered significant.

## 3 RESULTS

### 3.1 The characteristics of the patient and control groups

The median age of the patient group was 29 (24–34), and 24 (68%) of them were female. The HC group consisted of 15 (60%) females, and the median age was 31 (26–34.5) years. There was no statistically significant difference between the HCs and the patients in terms of sex and age (*p* = 0.493 and *p* = 0.494, respectively). The median age of onset of symptoms of patients with HAE was 11 (7–18) years, while the mean age at diagnosis was 22 ± 8.3 years. Thirty‐one (88.6%) patients had type 1 C1‐INH HAE and four (11.4%) patients had type 2 C1‐INH HAE. A family history of HAE was documented in 28 (80%) of the patients. Demographic and clinical characteristics of the patients are shown in Table 1.

**TABLE 1:** Demographic and clinical characteristics of the patients.

Age, years, median (IQR)	29 (24–34)
Sex, female, *n* (%)	24 (68.6)
Age at onset symptoms, years, median (IQR)	11 (7–18)
Symptom duration, years, median (IQR)	16 (12–22)
Age at diagnosis, years, mean ± SD	22 ± 8.3
Disease duration, years, median (IQR)	6 (4–11)
Family history, *n* (%)	28 (80)
Type of disease, *n* (%)	
Type 1 C1 INH HAE	31 (88.6)
Type 2 C1 INH HAE	4 (11.4)
Involvement site in attacks, *n* (%)	
Airways	15 (42.9)
Extremity	35 (100)
Abdomen	33 (94.3)
Head and neck	21 (60)
Genital	15 (42.9)
Disease severity score, median (IQR)	6 (4–11)
Attacks number per year, median (IQR)	18 (7–42)
C1‐inhibitor level at diagnosis, mg/dL, median (IQR)	5.88 (4.8–8)
C4 level at diagnosis, mg/dL, median (IQR)	4.1 (3–5.7)
Treatment strategy, *n* (%)	
Long‐term prophylaxis	15 (42.9)
Danazol	11 (31.4)
Tranexamic acid	4 (11.4)
On demand	22 (62.9)

Abbreviations: C1‐INH HAE, C1 inhibitor deficient hereditary angioedema; HAE, hereditary angioedema; IQR, interquartile range; *n*, number of patients; SD, standard deviation.

### 3.2 Flow‐mediated vasodilation in the patient and control groups

**FIGURE 1:**
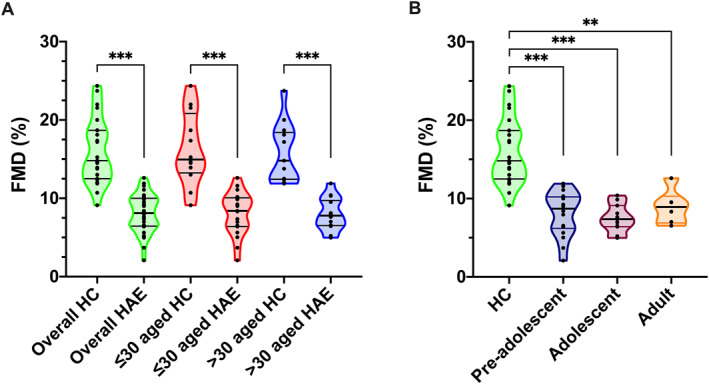
(A) Difference in FMD (%) between HC and patients with hereditary angioedema among overall, aged ≤30, and aged >30 subjects. (B) Difference in FMD (%) between HC and pre‐adolescent, adolescent, and adult‐onset HAE patients. All data show as median (IQR). Mann‐Whitney *U*, **p = 0.001, ***p < 0.001. FMD, flow‐mediated dilation; HAE, hereditary angioedema; HC, healthy control; IQR, interquartile range.

The diameter of the brachial artery at baseline was measured as 3.22 (2.88–3.54) mm in patients and 3.06 (2.7–3.5) mm in HCs (*p* = 0.280). The diameter of the brachial artery at the hyperaemic phase was measured as 3.43 (3.12–3.82) mm in patients and 3.54 (3.06–4.12) mm in HCs (*p* = 0.649). The percentage of FMD (FMD [%]) was found to be significantly lower in the patient group than in HCs (8.11 [6.46–10.0] versus 14.81 [12.5–18.68], *p* < 0.001) (Table 2). In individuals aged ≤30 years, the median FMD (%) was significantly lower in patients with HAE compared with HCs (8.4 [6.4–10] versus 14.95 [13.24–20.83], *p* < 0.001). The median FMD (%) were similar between the disease onset age groups which were pre‐adolescence (aged <12 years), adolescence (aged 12–18 years), and adult (aged >18 years) but all patient groups exhibited significantly decreased rates in comparison to the HCs (8.7 [6.2–10.2], 7.37 [6.41–9.11], and 8.92 [6.89–10.2] versus 14.81 [12.5–18.68]; *p* < 0.001, *p* < 0.001, and *p* = 0.001, respectively) (Figure 1).

**TABLE 2:** Flow‐mediated dilation of the brachial artery and brachial artery diameter at baseline and in response to hyperaemia in patients with HAE and HCs.

	HAE (*n* = 35)	Control (*n* = 25)	*p*‐value^a^
BAD‐baseline, mm, median (IQR)	3.22 (2.88–3.54)	3.06 (2.7–3.5)	0.280
BAD‐hyperaemia, mm, median (IQR)	3.43 (3.12–3.82)	3.54 (3.06–4.12)	0.649
FMD, %, median (IQR)	8.11 (6.46–10.0)	14.81 (12.5–18.68)	<0.001

Abbreviations: BAD, brachial artery diameter; FMD, flow‐mediated dilation; HAE, hereditary angioedema; IQR, interquartile range.

^a^ Mann‐Whitney *U*.

### 3.3 Associated factors with flow‐mediated vasodilatation in the patients

**FIGURE 2:**
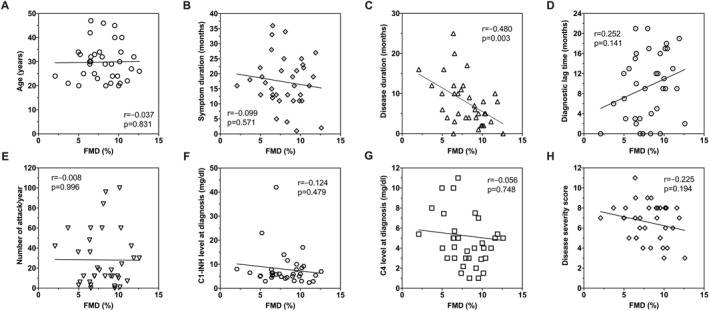
Correlation analysis between the percentage of flow‐mediated dilation and age (A), symptom duration (B), disease duration (C), diagnostic lag time (D), number of attacks per year (E), serum C1‐inhibitor level at diagnosis (F), serum C4 level at diagnosis (G), and disease severity score (H). Spearman's rho. C1‐INH, C1‐inhibitor; FMD, flow‐mediated dilation; *r*, correlation coefficient.

FMD (%) was 7.62 (6.54–10.18) in male patients, while it was 8.22 (6.42–9.78) in females (*p* = 0.137). There was no correlation between age and FMD (%) (*r* = −0.037, *p* = 0.831). A statistically significant negative correlation was found between disease duration and FMD (*r* = −0.480, *p* = 0.003), whereas no correlation was observed in terms of events per year and C4 and C1 inhibitor levels at diagnosis (*r* = −0.008, *p* = 0.966; *r* = −0.056, *p* = 0.748; *r* = −0.124, *p* = 0.479, respectively). No correlation was also found between disease severity score and FMD (%) (*r* = −0.225, *p* = 0.194) (Figure 2).

There was no difference in FMD (%) between the patients treated with and without danazol (9.52 [6.54–11.1] versus 8.03 [6.39–9.71], *p* = 0.252, respectively). Moreover, FMD (%) was 7.37 (6.54–10.18) in the patients with long‐term prophylactic treatment (danazol or tranexamic acid), and 8.22 (6.42–9.96) in the patients without long‐term prophylactic treatment (*p* = 0.390). A lack of sufficient subjects precluded a separate assessment of patients using tranexamic acid.

### 3.4 Carotid intima‐media thickness measurements in the patient and control groups

Left CIMT was 0.53 (0.48–0.58) mm, and right CIMT was 0.52 (0.49–0.59) mm in the patient group, while 0.55 (0.49–0.6) mm, and 0.56 (0.48–0.59) mm were measured in HC, respectively. There was no difference in left and right CIMT between the patient and control groups (*p* = 0.290, *p* = 0.594, respectively). The mean CIMT in the patient group was similar to the HC (0.52 [0.48–0.59] mm versus 0.53 [0.50–0.59], *p* = 0.504) (Table 3).

**TABLE 3:** Carotid intima‐media thickness measurements in patients with HAE and HCs.

	HAE (*n* = 35)	Control (*n* = 25)	*p*‐value^a^
L‐CIMT, mm, median (IQR)	0.53 (0.48–0.58)	0.55 (0.49–0.6)	0.290
R‐CIMT, mm, median (IQR)	0.52 (0.49–0.59)	0.56 (0.48–0.59)	0.594
Mean‐CIMT, mm, median (IQR)	0.52 (0.48–0.59)	0.53 (0.50–0.59)	0.504

Abbreviations: HAE, hereditary angioedema; IQR, interquartile range; L‐CIMT, left carotid intima‐media thickness; Mean‐CIMT, mean value of the left and right carotid intima‐media thickness; R‐CIMT, right carotid intima‐media thickness.

^a^ Mann‐Whitney *U*.

### 3.5 Transthoracic echocardiography findings in the patient and control groups

Transthoracic ECHO findings of the study subjects are presented in Table 4. These findings were similar between groups except for interventricular septal diameter (*p* = 0.046), although this small difference was not clinically significant.

**TABLE 4:** Transthoracic echocardiography findings in the patients with HAE and HCs.

	HAE (*n* = 35)	Control (*n* = 25)	*p*‐value^a^
LAVi, mL/m^2^, median (IQR)	22 (19–28)	20 (18–23.5)	0.106
LAV, mL/m^2^, median (IQR)	40 (33–46)	36 (30.5–42)	0.124
LAD, cm, median (IQR)	3.4 (3.2–3.5)	3.5 (3.2–3.6)	0.575
RAD, cm, median (IQR)	3.1 (2.9–3.3)	3.1 (2.85–3.35)	0.551
IVSD, cm, median (IQR)	1 (1–1.1)	1 (0.8–1.1)	0.046
PWD, cm, median (IQR)	0.9 (0.9–1.0)	0.9 (0.7–1.0)	0.123
LV mass index, g/m^2^, median (IQR)	84 (73–93)	74 (66.5–88.5)	0.115
LVEDD, cm, median (IQR)	4.5 (4.3–4.8)	4.5 (4.2–4.9)	0.928
LVESD, cm, median (IQR)	3 (2.8–3.1)	3 (2.65–3.1)	0.672
LVEF, %, median (IQR)	65 (59–71)	66 (63–69.5)	0.409
RVD‐mid, cm, median (IQR)	2.6 (2.5–2.7)	2.5 (2.3–2.7)	0.616
PAPs, mmHg, median (IQR)	25 (22–28)	25 (22–28.5)	0.976
E/A ratio, median (IQR)	1.3 (1.18–1.51)	1.44 (1.25–1.59)	0.251
E/e' ratio, median (IQR)	5.81 (5.3–6.37)	6.39 (4.49–6.17)	0.109
TAPSE, median (IQR)	2.1 (2–2.3)	2.2 (2–2.4)	0.171

Abbreviations: HAE, hereditary angioedema; IQR, interquartile range; IVSD, interventricular septum diameter; LAD, left atrial diameter; LAV, left atrial volume; LAVi, left atrial volume index; LV mass index, left ventricular mass index; LVEDD, left ventricule end diastolic diameter; LVEF, left ventricule ejection fraction; LVESD, left ventricle end systolic diameter; PAPs, systolic pulmonary artery pressure; PWD, posterior wall diameter; RAD, right atrial diameter; RVD‐mid, right ventricle mid diameter; TAPSE, tricuspid annular plane systolic excursion.

^a^ Mann‐Whitney *U*.

## 4 DISCUSSION

The current study made a substantial contribution to clinical practice and literature by demonstrating the presence of endothelial dysfunction in relatively young C1‐INH HAE patients without overt or subclinical atherosclerosis and additional risk factors. Furthermore, the study suggests a possible association between the presence of endothelial dysfunction and longer disease duration, although these warrants verification by further research. Given the inherited disorder nature of HAE, endothelial dysfunction would also be expected to be associated with age and symptom duration. This knowledge may lead us to consider HAE as a potential risk factor for endothelial dysfunction among other significant atherosclerotic risk factors and requires further attention in the management of HAE.

The endothelium, which is an active and dynamic barrier that continuously regulates the cardiovascular system, is a complex organ that responds to a variety of stimuli in the form of contraction or relaxation.^17^ Endothelial permeability is crucial in the pathogenesis of inflammation and the immune response.^18^ Paroxysmal Permeability Diseases (PPDs) are a group of disorders that are caused by or exacerbated by recurrent and transient alterations in endothelial permeability, without any ischaemic, degenerative, or inflammatory vascular damage. HAE, which is a clinical phenotype of PPDs, is characterised by recurrent episodes of swelling.^19^ The endothelium plays a crucial role in the pathogenesis of angioedema through the activation of the local contact—kallikrein/kinin system activation.^20^ Consequently, vasoactive mediators associated with endothelial function, and integrity of the endothelial functionality warrant further investigation.

Previous studies have demonstrated that the increased levels of vasoactive mediators, including arginine vasopressin, adrenomedullin (ADM), endothelin‐1 (ET1), impaired vascular endothelial integrity, and increased vascular permeability during attacks in patients with HAE. Several vasoactive mediators including vascular endothelial growth factor, angiopoietin‐2 (ANGPT2), increased in patients with more than 12 attacks per year. Platelet‐activating factor activity, which can directly modulate vascular permeability, remained elevated in patients with frequent attacks. ANGPT1 was increased and an inhibitor of vasodilating factors was decreased during the attack.^21^, ^22^, ^23^ Even during attack‐free period, the levels of endocan, vascular cell adhesion molecule, endothelial nitric oxide synthase (eNOS), and asymmetric dimethylarginine (ADMA) were found to be higher in HAE patients than in HCs.^11^, ^13^, ^23^ Elevated concentrations of ADMA, a potent inhibitor of NOS, are associated with impaired endothelium‐dependent vasodilation and an independent risk factor for atherosclerosis.^24^ Dynamics of vasoactive mediator levels during the oedematous and remission phases of the disease may temporally influence the endothelium. In this regard, the balance between regulatory and counter‐regulatory mediators may contribute to endothelial dysfunction at different levels in association with the frequency and duration of attacks. However, in the studies in which the presence of endothelial dysfunction at the molecular level was determined in HAE, the structural implications of the increase in vasoactive mediators were not demonstrated.

In a prior study conducted in HAE patients and controls with similar characteristics in terms of the causes of endothelial dysfunction showed that the CIMT was quite similar, whereas the coronary flow reserve (CFR) in the left anterior descending coronary artery and retinal thickness exhibited significant differences. The findings of this study provide evidence to suggest the possibility of microvascular endothelial dysfunction in patients with HAE.^25^ In another research, CIMT was found to be similar in line with our study.^26^

Nebenführer et al. reported no substantial difference in FMD assay between HAE patients and HCs indicating that there may be no association between HAE and endothelial dysfunction. Nevertheless, we believe that there are certain limitations to this study despite the valuable data. The study did not evaluate the level of HbA1c, CIMT, or the findings of ECHO to assess atherosclerosis, despite the similarity of the subject groups in terms of sex, age, and BMI. Furthermore, the patient's smoking status may have influenced endothelial function, potentially leading to misinterpretations of the study findings.^12^

In the study demonstrating endothelial dysfunction by different methods, the level of ADMA, which is a potent inhibitor of nitric oxide synthesis was measured, and the reactive hyperaemia index (RHI) were calculated by non‐invasive finger plethysmography. Patients with HAE had significantly lower RHI and higher ADMA compared with the HC group. No significant correlation was found between patient age, gender, age at disease onset, disease severity score, disease duration, and ADMA level and RHI.^11^ As with this study, our cohort also exhibited a female predominance. Attacks of HEA tend to be more frequent and severe in women than in men.^27^ Thus, endothelial dysfunction may manifest more prominently in that of Firinu et al. as well as in our study.^11^ In our study, no correlation was found between some of these parameters and endothelial dysfunction. However, the current study yielded a significant correlation between disease duration and FMD (%) supporting the hypothesis of endothelial dysfunction is related to the disease itself in HAE.

The association between endothelial dysfunction and HEA remains controversial, notwithstanding the accumulation of evidence. Variations in study designs and patient groups have the potential to influence the outcomes of studies.^28^ The present study evaluated patients without additional conditions associated with endothelial dysfunction, such as advanced age, smoking, obesity, diabetes mellitus, dyslipidaemia, and hypertension. Furthermore, we utilise transthoracic ECHO and CIMT measurements to exclude the presence of cardiac and subclinical atherosclerotic diseases. The results were compared with those of sex‐ and age‐matched HCs without risk factors for endothelial dysfunction. The study showed that FMD measurement of patients with HAE was significantly decreased compared with HCs, while CIMT was similar between groups. These findings indicate that endothelial dysfunction in HAE patients can be a consequence of the disease process rather than an atherosclerotic process. Moreover, the endothelial dysfunction can be identified and monitored by FMD, an easily applicable method, before the onset of overt or subclinical atherosclerotic diseases that may subsequently develop.

The dyslipidaemia effect of attenuated androgens, such as danazol, used for long‐term prophylaxis can be thought to be one of the potential causes of endothelial dysfunction in HAE.^12^ The long‐term use of danazol was found to be associated with dyslipidaemia and an increased risk for early atherosclerosis in patients with HAE.^29^ In previous studies, CIMT was found to be similar between patients with HAE treated with danazol and HC group.^30^, ^31^ In addition, the FMD measurements were also found to be similar between patients who were and were not under danazol.^25^ In the study in which CFR was evaluated, there was no evidence of an association between danazol use and CFR.^26^ The present study did not include patients with lipid profile disorders. A comparison of the data from patients who were and were not under danazol revealed no significant difference between FMD and CIMT measurements in this study. Therefore, it can be concluded that danazol may not cause endothelial dysfunction in the absence of a lipid profile disorder. In addition, the protective effects of tranexamic acid on the endothelium may have affected FMD (%) in the patient group.^28^ Further studies are required to elucidate the presence and mechanism of this possible effect.

Although the study revealed valuable information for the clinical practice and literature, it had some limitations. Vasoactive mediators related to endothelial dysfunction were not evaluated in blood samples. We were able to include a relatively small number of the subjects. HAE is a rare disease and we had to exclude some of the patients due to the presence of conventional atherosclerosis risk factors. The potential indirect or direct effect of some drugs such as danazol and tranexamic acid on the endothelium is an important factor for study design. Additionally, we were unable to include asymptomatic individuals who carry the genetic mutation, which restricts our ability to fully assess the relationship between endothelial dysfunction and disease duration. Including such individuals could have provided deeper insights into the variability of symptom onset and disease progression. Nevertheless, one of the major strengths of our study is the use of younger‐aged matched groups with adjustment for additional risk factors.

In conclusion, our study provides evidence that structural endothelial dysfunction occurs independently of atherosclerosis in HAE patients. Furthermore, it was demonstrated that endothelial dysfunction could be associated with a longer disease duration. We showed that patients with HAE are at risk of developing endothelial dysfunction from a young age and they could be susceptible to endothelial dysfunction, such as individuals with atherosclerosis. Consequently, prospective observation of these patients may be useful in terms of decreasing mortality and morbidity, given the potential for early detection and intervention in the development of cardiovascular diseases. Further studies are required to clarify the molecular mechanisms underlying endothelial dysfunction observed in HAE patients. Also, it is warranted to assess the effect of early endothelial dysfunction on mortality and morbidity of HAE by prospective follow‐up of patients.

## AUTHOR CONTRIBUTIONS

**Gokce Gul Atay Sensoy**: Conceptualization; investigation; writing—original draft; data curation. **Derya Baykız**: Data curation; investigation. **İlkim Deniz Toprak**: Data curation. **Deniz Eyice Karabacak**: Data curation. **Erdem Bektas**: Writing—original draft; writing—review and editing; visualization; formal analysis. **Derya Unal**: Data curation. **Aslı Gelincik**: Conceptualization; writing—review and editing; supervision. **Semra Demir**: Conceptualization; writing—review and editing; supervision.

## CONFLICT OF INTEREST STATEMENT

The authors declare no conflicts of interest.

## Supporting information

Table S1

## Data Availability

The data that support the findings of this study are available from the corresponding author upon reasonable request.
