# Supplementary material for: C1 inhibitor deficient hereditary angioedema is related to endothelial dysfunction in young adult and middle‐aged patients
Source: Clin Transl Allergy. 2025 Jun 23;15(6):e70076. doi: 10.1002/clt2.70076 (PMC12185901; doi:10.1002/clt2.70076)
Supplement: Supplementary file 1 — Table S1 [file CLT2-15-e70076-s001.docx]

**Appendix**

| **Supplementary Table 1.** Clinical severity score (cumulated 0–10 points) | |
| --- | --- |
| Age at onset 0–5 years | 3 points |
| Age at onset 6–10 years | 2 points |
| Age at onset 11–20 years | 1 point |
| Age at onset >20 years | 0 point |
| Skin oedema ever | 1 point |
| Painful abdominal oedema ever | 2 points |
| Laryngeal oedema ever | 2 points |
| Other clinical manifestations | 1 point |
| Long-term prophylaxis ever | 1 point |
